# A Wireless Sensor Network-Based Combustible Gas Detection System Using PSO-DBO-Optimized BP Neural Network

**DOI:** 10.3390/s25103151

**Published:** 2025-05-16

**Authors:** Min Zhou, Sen Wang, Jianming Li, Zhe Wei, Lingqiao Shui

**Affiliations:** School of Computer Science, Civil Aviation Flight University of China, Guanghan 618307, China; zmliqin@cafuc.edu.cn (M.Z.); chengnan@cafuc.edu.cn (S.W.); findz@cafuc.edu.cn (Z.W.); shuilingqiao23@cafuc.edu.cn (L.S.)

**Keywords:** wireless sensor network, combustible gas detection, condition monitoring, PSO-DBO-BP neural network, sensor array fusion, industrial safety

## Abstract

Combustible gas leakage remains a critical safety concern in industrial and indoor environments, necessitating the development of detection systems that are both accurate and practically deployable. This study presents a wireless gas detection system that integrates a gas sensor array, a low-power microcontroller with Zigbee-based communication, and a Back Propagation (BP) neural network optimized via a sequential hybrid strategy. Specifically, Particle Swarm Optimization (PSO) is employed for global parameter initialization, followed by Dung Beetle Optimization (DBO) for local refinement, jointly enhancing the network’s convergence speed and predictive precision. Experimental results confirm that the proposed PSO-DBO-BP model achieves high correlation coefficients (above 0.997) and low mean relative errors (below 0.25%) for all monitored gases, including hydrogen, carbon monoxide, alkanes, and smog. The model exhibits strong robustness in handling nonlinear responses and cross-sensitivity effects across multiple sensors, demonstrating its effectiveness in complex detection scenarios under laboratory conditions within embedded wireless sensor networks.

## 1. Introduction

With the accelerating pace of global industrialization, especially in high-risk sectors such as petrochemicals, coal mining, and natural gas transportation, combustible gases have become integral not only to industrial productivity and energy supply, but also to the safety and sustainability of modern urban infrastructure. These gases are extensively used not only as fuels and raw materials in various production processes, but they have also become indispensable components of contemporary infrastructure and transportation systems. However, the frequency of flammable gas leakage incidents has significantly increased in recent years, leading to catastrophic consequences, including human casualties, severe property loss, and long-term environmental degradation. For instance, gases like methane and hydrogen can easily form explosive mixtures with air, posing a high risk of detonation upon exposure to ignition sources, while carbon monoxide leaks are notorious for causing acute poisoning and even fatalities.

Improving the detection accuracy and response speed of flammable gas leakage, as well as the early detection of potential gas leakage hazards, have become important issues in all walks of life. Efficient monitoring and early warning technologies for combustible gas leakage have gradually become the focus of social attention, driving the development of multi-sensor fusion detection [1], intelligent early warning systems [2], and intelligent gas sensing technology [3], in order to improve the detection accuracy, response speed, and environmental adaptability of gas leakage. Traditional gas detection methods, such as catalytic combustion and semiconductor sensors [4,5], often fall short in complex industrial environments due to limited sensitivity, poor selectivity, and susceptibility to environmental interference.

To address these limitations, researchers have increasingly turned to solutions that combine multi-sensor arrays with neural network-based learning frameworks, which enable robust and intelligent gas detection under dynamic conditions. The introduction of neural network technology [6], especially deep learning technology [7], enables gas detection to automatically extract features and carry out non-linear mapping through the learning and optimization of a large number of complex data, thus overcoming the problem of inaccurate detection caused by the interference of environmental factors in traditional methods. Multi-sensor array technology [8] can improve the accuracy of gas concentration measurement by real-time acquisition and fusion of multiple gas sensors. By analyzing the output data of multiple sensors, the sensor array can reduce the error and uncertainty caused by a single sensor and improve the robustness and reliability of the system.

With the rapid development of artificial intelligence technology, the combustible gas detection method based on neural networks has shown great potential in the field of gas detection. At present, many researchers have combined neural networks and gas sensor technology to develop a variety of high-precision and intelligent gas detection systems. For example, algorithms such as deep neural networks (DNN) and convolutional neural networks (CNN) can be applied to the data processing of gas sensor arrays [9], which can automatically extract effective features from large-scale data, and not only improve the detection accuracy, but also realize simultaneous detection of multiple gas mixtures.

Although neural network-based gas detection algorithms perform well in many ways, there are still some problems that need to be solved. On the one hand, traditional neural network models can be relatively complex and computationally intensive, which may limit their applicability in scenarios with strict real-time constraints. However, recent advances in lightweight neural network architectures such as MobileNet [10] and ShuffleNet [11] have significantly improved the feasibility of deploying deep learning models on edge devices. Supported by TinyML [12] frameworks and model compression techniques, these networks can run efficiently on microcontrollers and embedded platforms like STM32 [13] and Raspberry Pi Pico [14], enabling fast and energy-efficient gas concentration inference.

These advancements provide a promising foundation for deploying intelligent gas detection models in real-world, low-power environments, which aligns with the motivation of this study. On the other hand, how to choose the appropriate algorithm and model structure to deal with the complex and changeable detection environment is still a problem worthy of further study.

The main contributions and novelties of this study are threefold. First, we propose a hybrid optimization approach that sequentially combines Particle Swarm Optimization (PSO) and Dung Beetle Optimization (DBO) to improve both convergence speed and local search accuracy in training a Back Propagation (BP) neural network for gas concentration prediction. Second, this study integrates this model into a practical wireless sensor network (WSN) architecture featuring decentralized data acquisition using Zigbee-based MEMS sensor nodes. Third, we highlight the model’s robustness in handling cross-sensitivity among sensors and nonlinear interactions in multi-gas detection tasks, with validation through laboratory-scale data. These aspects distinguish the proposed method from existing works, which typically focus either on algorithmic improvement or hardware integration but rarely both.

The remainder of this paper is organized as follows: Section 2 reviews related work and introduces the core methodologies, including BP, PSO, and DBO. Section 3 presents the design of the hybrid PSO-DBO-BP model. Section 4 details the experimental setup and results. Section 5 and Section 6 discuss the findings and conclude this study.

## 2. Related Work and Methods

In recent years, neural networks have received significant attention in the field of gas detection. Researchers have conducted extensive studies on gas classification, concentration estimation, and real-time monitoring, leading to notable advancements in both algorithmic performance and practical deployments. With the rapid development of deep learning technologies, neural network-based gas detection algorithms have demonstrated excellent performance in nonlinear signal processing, adaptive feature extraction, and data fusion [15,16,17], contributing considerably to improvements in detection accuracy, anti-interference capability, and real-time prediction.

Advanced neural network models have been widely applied in gas detection tasks, particularly those involving multi-sensor array processing and complex environmental gas recognition. Deep neural networks (DNN), convolutional neural networks (CNN), Back Propagation (BP) networks, and radial basis function (RBF) networks have all demonstrated strong capabilities, and electronic nose (E-nose) systems that simulate the human olfactory mechanism have made notable progress in recent developments [18,19,20,21]. These systems combine multiple gas sensors with DNN and CNN models to perform feature extraction and pattern recognition, enabling precise identification of complex gas mixtures.

Furthermore, ref. [22] proposed an optimized PSO-BP neural network for assessing safety risks in underground coal mining environments. This model achieved a high correlation coefficient, demonstrating the practical feasibility of PSO-BP in nonlinear prediction tasks. However, this approach lacked hybrid optimization strategies and did not address the challenges of distributed sensing, which are crucial for real-time, edge-based gas monitoring tasks considered in this work. Ref. [23] designed an embedded assistive system for visually impaired individuals using a MobileNet model optimized via gradient PSO. Their system ran in real-time on a Raspberry Pi board and highlights the growing potential of deploying lightweight, optimized neural networks on microcontrollers. Although not applied to gas detection, this framework supports the direction adopted in our study, namely, integrating intelligent optimization with embedded sensing platforms.

Deep learning algorithms have also been deployed in smart inspection robots and wireless sensor networks (WSNs) for efficient detection of combustible gases such as methane, hydrogen, and carbon monoxide in complex environments [24]. Furthermore, industrial intelligent gas detection systems combining reinforcement learning and adaptive optimization methods have been developed by several laboratories and enterprises, improving system stability and generalization [25].

It is worth noting that recent advances in lightweight neural network architectures have made deployment on edge devices increasingly feasible. Models such as MobileNet, ShuffleNet, and SqueezeNet utilize techniques like depthwise separable convolutions, network pruning, and parameter compression to substantially reduce model size and computational complexity. With the aid of TinyML frameworks, these models can operate efficiently on microcontroller-based platforms such as STM32, ESP32, and Raspberry Pi Pico, enabling real-time inference. Existing studies have shown that compressed CNNs can achieve gas concentration classification with inference latency under 200 ms on such devices, providing a practical foundation for building low-power, real-time wireless monitoring systems. These findings indicate that the traditional view of neural networks as computationally intensive and unsuitable for deployment is no longer consistent with the current state of model development.

Ongoing research has also focused on optimizing network structures, improving training efficiency, and reducing computational complexity, leading to progress in small-sample learning and model generalization. For example, some studies have proposed techniques such as adaptive learning rates, momentum terms, and swarm intelligence optimization algorithms to improve the convergence and accuracy of BP networks [26,27]. CNNs have also shown outstanding advantages in extracting spatiotemporal features from high-dimensional sensor data, reducing the reliance on manual feature engineering [28,29]. In addition, recurrent neural networks (RNN) and their variants such as long short-term memory (LSTM) and gated recurrent units (GRU) have been applied in time-series modeling for gas leak forecasting, improving both timeliness and accuracy [30,31].

Despite significant progress, neural network-based gas detection systems still face several challenges. First, model training typically relies on large quantities of high-quality labeled data, which can be costly and difficult to obtain. Second, in time-critical environments, some deep models may impose high computational overhead, presenting challenges for deployment. However, it should be emphasized that with the development of lightweight neural architectures and edge deployment technologies, neural networks can now achieve real-time performance on resource-constrained platforms. Therefore, balancing detection accuracy, model complexity, and system constraints remains a key direction for future research in this field.

Building upon the strengths and limitations identified in prior work, this study proposes a hybrid PSO-DBO-BP neural network to improve gas concentration prediction accuracy and training efficiency. Unlike most existing studies that focus solely on algorithmic development or hardware deployment, our approach integrates intelligent optimization with wireless sensor networks for practical, embedded applications. The following sections present the individual components of the model and explain how they are combined to achieve enhanced performance and deployability.

### 2.1. BP Neural Network

**Figure 1:**
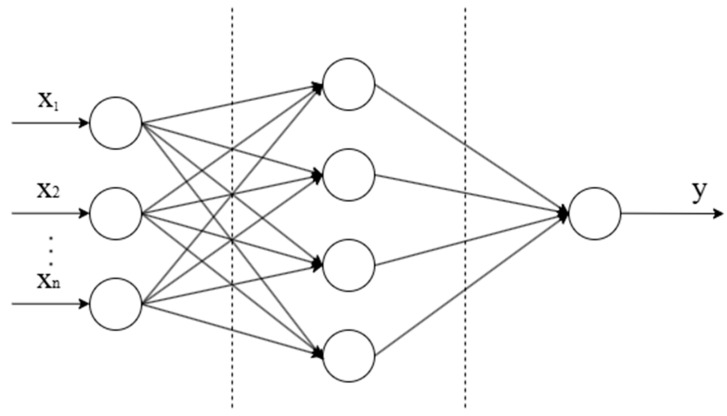
BP neural network architecture diagram.

The BP neural network is a common multilayer feedforward neural network, which is widely used in function approximation, pattern recognition, and other fields. The core idea is to use the error Back Propagation algorithm to minimize output errors and optimize model performance by constantly adjusting network weights and bias terms [32,33]. The basic structure of the BP neural network is shown in Figure 1.

The BP neural network is composed of an input layer, a hidden layer, and an output layer, and each layer is connected by weights. The input layer is responsible for receiving external information, the hidden layer carries out nonlinear mapping through activation functions, and the output layer ultimately generates prediction results [32]. The learning process of the network is essentially the learning of the mapping relationship between input and output, which can automatically extract features from the training data and continuously optimize model performance.

The neurons in the input layer receive the external signal, and the hidden layer sums the weighted input signal and passes it to the next layer through the activation function. The output layer generates the predicted value based on the final calculation result.

### 2.2. Particle Swarm Optimization

Particle Swarm Optimization (PSO) is a swarm intelligence-based optimization algorithm proposed by Kennedy and Eberhart in 1995, inspired by the foraging behavior of birds. This algorithm seeks the global optimal solution in a collaborative manner by simulating the movement of individuals in the search space [34]. In PSO, each candidate solution is called a “particle”, which flies at a certain speed in the search space and updates its position by interacting with its own historical experience and the experience of other particles, and finally finds the global optimal solution [35]. Each particle has two main properties: position and velocity. Position represents the coordinates of the current solution, while velocity controls the direction and step size of the particle’s movement. The motion of a particle is affected by two factors: one is its own historical optimal position (Personal Best, pbest) and the other is the historical optimal position of the whole group (Global Best, gbest). The PSO speed update formula is as follows:

(1) $$v_{i}^{t+1}=\omega v_{i}^{t}+c_{1}r_{1}\left(pbest_{i}-x_{i}^{t}\right)+c_{2}r_{2}\left(gbest-x_{i}^{t}\right)$$

In the formula, ω is the inertial weight, which controls the global search and local search ability of particles, $c_{1}$ and $c_{2}$ are learning factors (acceleration constants), which are used to adjust the dependence of particles on their own experience and group experience, $r_{1}$ and $r_{2}$ are random numbers between [0, 1], and randomness is introduced to enhance the search ability.

The execution steps of the PSO algorithm are as follows: First, the position and velocity of a group of particles are initialized, and the fitness value of each particle is calculated to find the individual historical optimal position $pbest_{i}$ and the global optimal position gbest. Then, the speed and position of the particles are iteratively updated, the fitness value is calculated after each update, the current solution is compared with the historical optimal solution, and the individual optimal and the global optimal are updated. This process continues until the maximum number of iterations is reached, or convergence to a satisfactory error range [36].

PSO has some obvious advantages, such as simple calculation, easy implementation, no need for gradient information of the objective function, and is especially suitable for high-dimensional nonlinear optimization problems. But PSO also has some shortcomings, such as being easy to fall into local optima, and convergence speed being greatly affected by parameter settings. In order to solve these problems, researchers have proposed many improved versions, such as adaptive weight PSO [37], hybrid PSO [38], combined with other optimization algorithms, etc.

### 2.3. Dung Beetle Optimization

Dung Beetle Optimization (DBO) is an intelligent optimization algorithm based on the biological behavior characteristics of dung beetles. By simulating the behavior of dung beetles in the natural environment, such as foraging, dung ball rolling, navigation, and reproduction, the dung beetle realizes the coordinated optimization of global search and local search [39].

The core mechanism of this algorithm includes ball rolling, reproduction, foraging, and stealing, in which the ball rolling process simulates the behavior of dung beetles constantly adjusting the path while rolling the dung ball, enabling individuals to conduct global exploration in the vast search space to find the potential optimal solution [40]. The reproduction mechanism is used to simulate the expansion of dung beetle populations and increase population diversity by generating new individuals, thereby avoiding the search for local optima [41]. The foraging mechanism simulates the fine search of dung beetles around dung piles, enabling individuals to further optimize solutions and improve convergence accuracy when approaching the target area [42]. The stealing mechanism simulates that some dung beetles directly seize the dung balls of other dung beetles to speed up the search process and improve optimization efficiency [43]. According to [40,41,42,43,44,45,46], the process of DBO can be described as follows.

1. Rolling dung beetle position update

Dung beetles search for a global optimal solution in their natural environment by rolling dung balls to find suitable storage sites. DBO carries out global search through the rolling ball process, that is, each individual moves closer to the optimal individual, while adding random perturbations to avoid falling into the local optima. The location of an individual is updated as follows:

(2) $$X_{i}^{t+1}=X_{i}^{t}+\alpha \left(X_{\mathrm{best}}^{t}-X_{i}^{t}\right)+\beta \mathrm{randn}\left(0,1\right)$$

where $X_{best}^{t}$ represents the position of the optimal individual in the current population, α controls the global search intensity, β is the random disturbance factor used to enhance the search diversity, $randn\left(0,1\right)$ represents the random number conforming to the normal distribution, helping the individual to jump out of the local optima.

2. Location update of breeding dung beetles

In nature, dung beetles breed to expand their population and improve their competitiveness. DBO uses this mechanism to generate new solutions to enhance the diversity of search and avoid falling into local optima during optimization. The new individual is generated by the current optimal individual variation, and its position is calculated as follows:

(3) $$X_{new}=X_{best}+\gamma \cdot \left(X_{rand}-X_{best}\right)$$

where $X_{rand}$ represents randomly selected individuals, and γ is the control factor that determines the deviation degree of new individuals. This process can help the population to form a richer distribution in the search space and improve the exploration ability of the algorithm.

3. Location update of foraging dung beetles

Dung beetles forage around dung mounds to find the best quality balls. In DBO, this process is used for local search, allowing individuals to further adjust their position as they approach the optimal solution to improve optimization accuracy. Individuals can be updated as follows:

(4) $$X_{i}^{t+1}=X_{i}^{t}+\lambda \left(X_{local}^{t}-X_{i}^{t}\right)$$

where $X_{local}^{t}$ is a random solution around the current individual, and λ controls the local search range. This mechanism enables DBO to have stronger local optimization ability, further fine-tune the solution after global search, and improve the convergence effect.

4. Thieving dung beetle location update

In nature, some dung beetles directly take the dung balls of other individuals to get to resources faster. In DBO, stealing behavior is used to accelerate convergence so that individuals quickly approach the optimal solution in the late optimization period. Individuals can be updated as follows:

(5) $$X_{i}^{t+1}=X_{i}^{t}+\rho \left(X_{best}^{t}-X_{i}^{t}\right)$$

In the formula, ρ is the stealing factor, which controls the speed of the individual moving to the optimal solution. This mechanism makes search more efficient, reduces unnecessary substitution in the later stages of optimization, and improves the overall convergence speed.

Compared with the traditional gradient optimization methods, DBO does not depend on the gradient information of the objective function, and has strong global search ability and local optimization ability, which is suitable for complex, multidimensional, and nonlinear optimization problems. Because of this characteristic, DBO has been widely used in neural network training and other fields, showing strong advantages in solving complex optimization problems.

To further enhance the training performance of the BP neural network, in this work, the PSO and DBO algorithms are applied using a sequential hybrid strategy to train the BP neural network. Specifically, PSO is used in the first stage to perform a global search, initializing the weights and thresholds of the network. This step helps avoid poor local minima by efficiently exploring a broader solution space.

Subsequently, the solution obtained from PSO is passed to the second stage, where the DBO algorithm performs adaptive local optimization. The DBO algorithm enhances convergence stability and precision by simulating the multi-phase foraging behaviors of dung beetles.

This two-stage optimization scheme enables the proposed model to benefit from the rapid convergence of PSO and the refined exploitation of DBO, improving prediction performance in terms of both accuracy and generalizability.

In selecting optimization algorithms for this study, we considered several well-known alternatives, including the Genetic Algorithm (GA), Whale Optimization Algorithm (WOA), and Grey Wolf Optimizer (GWO), all of which have been widely used for neural network parameter tuning [47,48,49]. However, these methods either require extensive parameter tuning, suffer from slow convergence, or show instability in small-sample regression tasks.

In contrast, PSO is recognized for its rapid global search efficiency, while DBO introduces multi-phase local refinement to help avoid premature convergence. These characteristics make PSO and DBO particularly well suited for our sequential hybrid strategy, which aims to balance exploration and exploitation in a compact dataset scenario.

Although other algorithms were not implemented in this version, we acknowledge their relevance and plan to include them in future extended comparative studies.

### 2.4. Hardware System and Experimental Data

#### 2.4.1. Hardware System

**Figure 2:**
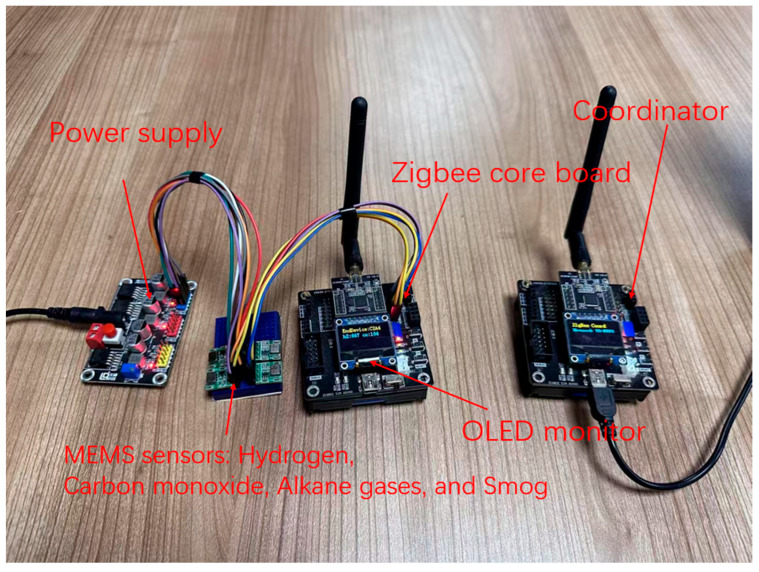
Hardware system.

The hardware detection system designed in this study includes the following modules: a micro-controller module, Zigbee wireless communication module, gas sensor array module, OLED display module, and power module. The gas sensor array module collects gas concentration information through the I^2^C protocol and transmits the data to the micro-controller module. The micro-controller module works in conjunction with the Zigbee wireless communication module to transmit the concentration information collected by the gas sensor array of each terminal node to the coordinator, which then transmits the data to the upper computer through the serial port. Finally, in the upper computer, the gas detection accuracy enhancement algorithm is used to analyze and process the transmitted data. Figure 2 shows a working diagram of the hardware inspection system, which clearly shows the actual configuration and layout of each module.

To support energy-efficient operation, the system incorporates low-power design principles. Each sensor node uses a CC2530 microcontroller (Texas Instruments Incorporated, Shenzhen, China) with an integrated Zigbee transceiver (Mouser Electronics, Inc., Mansfield, TX, USA) that supports multiple sleep modes. The LM2596T-3.3 DC-DC converter (Texas Instruments Incorporated, Shenzhen, China) ensures a stable 3.3 V power supply with high efficiency. Based on the sampling rate of one transmission every 0.5 s and typical operating profiles, the average current draw per node is estimated to be under 25 mA. This corresponds to approximately 250 mWh of energy usage per hour per node when powered by a standard 3.3 V lithium battery, making the system suitable for extended runtime applications.

The system utilizes a gas sensor array composed of four metal oxide semiconductor (MOX) sensors, which are known for their effectiveness in detecting various gases. These sensors include the following:

- JND103—H_2_
- JND104—CO
- JND109—C_n_H_2n+2_
- JND115—Smog

These sensors are produced by well-known manufacturers such as Hanwei Electronics Group Co., Ltd. (Zhengzhou, China), a company widely recognized for its reliable and cost-effective gas sensing solutions used in environmental and industrial monitoring systems.

#### 2.4.2. Experimental Data Acquisition

The combustible gas data acquisition system designed in this study is based on multi-sensor array technology, combined with modern signal acquisition and processing methods, to realize the synchronous monitoring of the concentration of hydrogen, carbon monoxide, alkane gas, and smog. Through the fusion of various gas sensors, the system can obtain the concentration data of different gases efficiently and accurately. The sample data acquisition process of this study was carried out in different gas environments, including normal air environment, gas leakage environment, combustion environment, etc. Since the change in gas concentration is greatly affected by environmental factors, the method of periodic and multi-point sampling was adopted in the data collection process to improve the representativeness of data.

The sampling period is to record data every 0.5 s, and 100 groups are required to obtain gas data in different environments. The sensor data are stored in a standard format, and the output data are shown in Table 1. Due to the large amount of data, only part of the data are shown.

In the process of data acquisition, the gas sensor array interacts with the micro-controller through the I^2^C protocol, and transmits the collected sensor data to the upper computer through the UART serial port at a sampling period of 0.5 s, and stores it in a standard format. The data collected each time include time stamps and H_2_, CO, C_n_H_2n+2_, and smog gas concentration values at the corresponding time. If the sensor reads abnormally in an air environment (for example, much higher than normal), it is re-initialized.

In addition to the basic acquisition cycle and sensor layout, the wireless communication and data processing mechanisms also play an essential role in ensuring the reliability and responsiveness of the overall detection system.

The wireless data transmission of the system is based on the Zigbee protocol (IEEE 802.15.4), using the CC2530 microcontroller with an integrated 2.4 GHz transceiver. Each terminal node transmits collected data to a coordinator every 0.5 s. The theoretical maximum node support of the system is 10, while the current deployment uses four sensor nodes to train and test the model.

Sensor readings are transferred from the MEMS sensor array to the microcontroller via the I^2^C protocol, then sent to the PC host via UART, and processed using a custom MATLAB 2021b App Designer interface. Data processing includes timestamp labeling, cyclic buffer filtering, normalization, and inference using the trained PSO-DBO-BP model.

Although detailed benchmarking of communication latency and per-minute data capacity was not the focus of this study, each node transmits approximately 80 bytes per sampling leading to an estimated data load of 9600 bytes/min/node.

Cross-node consistency was monitored manually. Across multiple trials in identical test environments, sensor nodes showed mean reading differences under 2% for all gas types. We understand the importance of reporting statistical variation metrics such as standard deviation and will include this in future deployment-scale experiments under consistent environmental conditions.

#### 2.4.3. Calibration and Experimental Conditions

To ensure the reliability of the gas concentration labels used in model training, each sample was collected with the aid of high-precision reference sensors placed alongside the experimental gas sensor array. The reference devices include TGS813-series detectors (Figaro Engineering Inc., Shanghai, China). During each acquisition cycle, both the target sensor array and reference sensors were co-located in a sealed test chamber to ensure exposure to identical gas concentrations. The outputs of these calibrated detectors were recorded as the ground truth to train and validate the BP neural network model.

Calibration tests were performed under ambient indoor laboratory conditions, with temperature maintained around 23 °C and relative humidity approximately 55%. No intentional humidity variation was introduced, but temperature and humidity readings were monitored and passed through filtering circuits and normalization pipelines before model input.

The smog environment was generated by controlled combustion in the sealed chamber. This procedure produced stable particulate concentrations and trace gases, simulating a realistic indoor polluted scenario. A total of 100 group data sample sets were collected during these experiments. Of these, 70 sets were randomly selected for model training, and the remaining 30 were used for validation.

The high-precision gas detectors used as reference instruments for gas concentration measurements include the following:

- CO analyzer for carbon monoxide measurement;
- H_2_ analyzer for hydrogen measurement;
- C_n_H_2n+2_ analyzer for alkane measurement;
- Particulate and air quality analyzer for smog measurement.

These analyzers are laboratory-grade devices designed for accurate and reliable gas measurements, ensuring precise calibration of the experimental setup. The values from these reference detectors were used to calibrate the experimental gas sensor array and to ensure that the results from the MOX-based sensors were consistent with actual gas concentrations.

While these reference sensors are commonly used in environmental and industrial monitoring systems, they were calibrated against known concentrations of gas and used to validate the predictions made by the PSO-DBO-BP model in the experiments.

## 3. PSO-DBO-BP Detection Method

In the combustible gas detection in this study, the prediction performance of the BP neural network is highly dependent on the optimization of weights and thresholds. Combining the global search capability of PSO and the local optimization capability of DBO, the PSO-DBO-BP strategy not only speeds up the convergence speed, but also improves the final optimization accuracy, making the combustible gas detection more efficient and stable.

In this study, the concentrations of hydrogen, carbon monoxide, alkanes, and smog gases detected by sensor array are taken as input characteristics, and the real concentrations measured by a high-precision single gas detector close to the true concentration value are taken as output targets. A BP neural network model based on the joint optimization of PSO and DBO is constructed to achieve accurate prediction of combustible gas concentrations.

In the actual detection environment, due to the cross-sensitivity of the gas sensor, it is not only affected by the target gas, but also interfered by other co-existing gases, resulting in complex nonlinear characteristics of the output response signal. The specific mathematical relationship can be expressed as follows:

(6) $$\left\{\begin{array}{c} y_{1}=f_{1}(r_{1},r_{2},r_{3},r_{4})\\ y_{2}=f_{2}(r_{1},r_{2},r_{3},r_{4})\\ y_{3}=f_{3}(r_{1},r_{2},r_{3},r_{4})\\ y_{4}=f_{4}(r_{1},r_{2},r_{3},r_{4}) \end{array}\right.$$

where $f_{i}(\cdot )$ represents the unknown nonlinear mapping function, $y_{i}$ is the response value of sensor *i*, and $r_{i}$ is the true concentration of the *i*th gas. Since this functional relationship is complicated and difficult to analyze directly, this study uses a BP neural network for nonlinear fitting to learn the mapping relationship between the output of the input gas sensor and the real gas concentration.

Through data acquisition under different environmental conditions (such as leakage and combustion), this paper constructs a total of 100 group data samples (a total of 1600 data points). Each set of data contains 4 input values and 4 output values, in which the input is the concentration data of 4 kinds of gases detected by the designed sensor array ($y_{1}$, $y_{2}$, $y_{3}$, $y_{4}$), and the output is the true concentration values obtained by 4 high-precision single gas detectors $(r_{1},r_{2},r_{3},r_{4}$). To ensure data diversity and generalization ability, 100 sets of data were divided into training sets (70 sets) and test sets (30 sets) by random sampling. Due to the difference in dimension and measurement range between sensor data, the input data are first normalized before network training.

In this study, the basic structure of BP neural network is constructed: three layers of feed-forward neural network, including input layer, hidden layer, and output layer. The number of nodes in the input layer is 4 (corresponding to the output of 4 kinds of sensors), and the number of nodes in the output layer is also 4, respectively, to output the predicted concentration of 4 kinds of gases. The hidden layer uses sigmoid function for nonlinear feature extraction, and the output layer uses linear transfer function.

Traditional BP networks rely on gradient descent for optimization, but gradient descent is easy to fall into local optima and sensitive to initial weights, resulting in slow training convergence. In contrast, PSO-DBO-BP adopts intelligent optimization strategy, which does not need to calculate gradient information, and can find the optimal weight in a wider search space, improving the stability and generalization ability of the model.

In order to improve the optimization effect, Particle Swarm Optimization (PSO) was first introduced to optimize the weight and bias of BP neural network. PSO has good global search ability and can quickly find the optimal weight in a large search space to avoid BP falling into the sub-optimal solution due to improper random initialization.

However, PSO is prone to premature convergence and lack of local search ability. In order to further optimize the local search ability of PSO, the Dung Beetle Optimization algorithm is introduced to adjust the initial solution provided by PSO by using its dynamic search mechanism. By simulating the ball rolling behavior of dung beetles, DBO achieves a smooth transition from global exploration to local optimization, making weight adjustment more adaptable and improving convergence accuracy and stability. Since BP neural network optimization is a single-objective continuous optimization problem, which does not involve population evolution and resource allocation, this study only adopts DBO’s rolling ball mechanism, instead of breeding, foraging, and other mechanisms, in order to improve optimization efficiency and reduce computational complexity.

In the DBO optimization process, the ownership weight and bias of the BP neural network are regarded as the position vector X in the DBO algorithm, and the optimal parameter combination is sought through continuous iteration to minimize the mean square error loss function of the network output. The specific process includes the following:

1. Initialize the BP neural network

In the initialization stage, the structure of the BP neural network is determined first, including the number of neurons in input layer, hidden layer, and output layer. The weights and bias are then initialized randomly. Because BP network training is very sensitive to the initial value of weights, reasonable initialization can accelerate convergence and avoid falling into local optima. The initialization process can be expressed as follows:

(7) $$W_{ij}^{0},B_{j}^{0}=X_{min}+rand\left(0,1\right)\times \left(X_{max}-X_{min}\right)$$

where $W_{ij}^{0}$ and $B_{j}^{0}$ respectively represent the weights and biases of the ith input to the jth hidden layer neuron, $X_{min}$ and $X_{max}$ are the upper and lower bounds of the weights, and $rand\left(0,1\right)$ generates random numbers to ensure diversity in the initialization process.

2. PSO is adopted for global optimization

In the PSO optimization process, each particle p represents a set of weights and biases of the BP network, and the position matrix of the particle swarm is defined as follows:

(8) $$X^{p}=\left[W_{ij}^{p},B_{j}^{p}\right]$$

The motion of the particle in the search space is determined by the velocity update equation and the position update equation:

(9) $$V^{p}\left(t+1\right)=\omega V^{p}\left(t\right)+c_{1}r_{1}\left(P_{\mathrm{best}}^{p}-X^{p}\left(t\right)\right)+c_{2}r_{2}\left(G_{\mathrm{best}}-X^{p}\left(t\right)\right)$$

(10) $$X^{p}\left(t+1\right)=X^{p}\left(t\right)+V^{p}\left(t+1\right)$$

where $V^{p}$ is the particle velocity, $P_{best}^{p}$ is the historical optimal position of the particle, $G_{best}$ is the global optimal position, ω is the inertia weight, $c_{1},c_{2}$ are the acceleration factor, $r_{1},r_{2}$ are random numbers, which are used to enhance the search diversity.

After PSO global search, the initial weights of the BP neural network have been optimized. In order to further improve the precision of weight optimization and the convergence stability of the network, DBO is introduced on the basis of PSO results for local optimization.

3. Initialize DBO parameters

In the DBO optimization process, each dung beetle individual represents the weights and biases of a set of BP neural networks, and is initialized on the basis of the optimization results calculated by PSO. The initialization formula is as follows:

(11) $$X_{i}^{0}=X_{\mathrm{PSO}}+\gamma \cdot rand\left(0,1\right)\cdot \left(X_{\max}-X_{\min}\right)$$

where $X_{PSO}$ is the initial optimal weight calculated by PSO, and γ is the disturbance factor (used to maintain population diversity and avoid all individuals being concentrated in the same point).

4. DBO ball search optimization

In each iteration, individual dung beetles searched based on the optimal solution provided by the PSO and further optimized the weights and biases on a local scale through a rolling search mechanism, while using random perturbations to enhance the search capability. The updated formula is as follows:

(12) $$X_{i}^{\left(t+1\right)}=X_{i}^{t}+\alpha \cdot \left(X_{\mathrm{best}}^{t}-X_{i}^{t}\right)+\beta \cdot randn\left(0,1\right)\cdot \left(X_{\max}-X_{\min}\right)$$

where $X_{best}^{t}$ is the current optimal individual in the DBO iteration process (non-PSO global optimal).

5. Calculation fitness (loss function)

In order to ensure the consistency of optimization objectives, DBO adopts the same fitness calculation method as PSO, namely mean square error (MSE), to measure the optimization effect.

6. Update the optimal solution

After each iteration, the current optimal individual is selected through fitness evaluation, and the global optimal solution is updated. The updated formula is as follows:

(13) $$X_{best}^{t+1}=\arg minF\left(X_{i}^{t+1}\right),i=1,2,\ldots ,N$$

where $X_{best}^{t+1}$ represents the optimal solution after iteration *t* + 1 round. Through continuous iterative optimization, the optimal solution gradually approaches the global optimal.

7. Suspension condition

When the maximum number of iterations $T_{max}$ or fitness convergence is reached, the algorithm terminates and outputs the current optimal solution:

(14) $$\left|F\left(X_{best}^{t+1}\right)-F\left(X_{best}^{t}\right)\right|<\epsilon \;or\;t>T_{max}$$

where ϵ is the set fitness convergence threshold, $T_{max}$ is the maximum number of iterations, and t is the current number of iterations.

8. Output optimal weight and bias

After the optimization process is completed, the optimal weight and bias of BP neural network are finally output, which can be used for subsequent model training and combustible gas concentration prediction. The final output formula is as follows:

(15) $$W_{optimal},B_{optimal}=X_{best}^{T_{final}}$$

**Figure 3:**
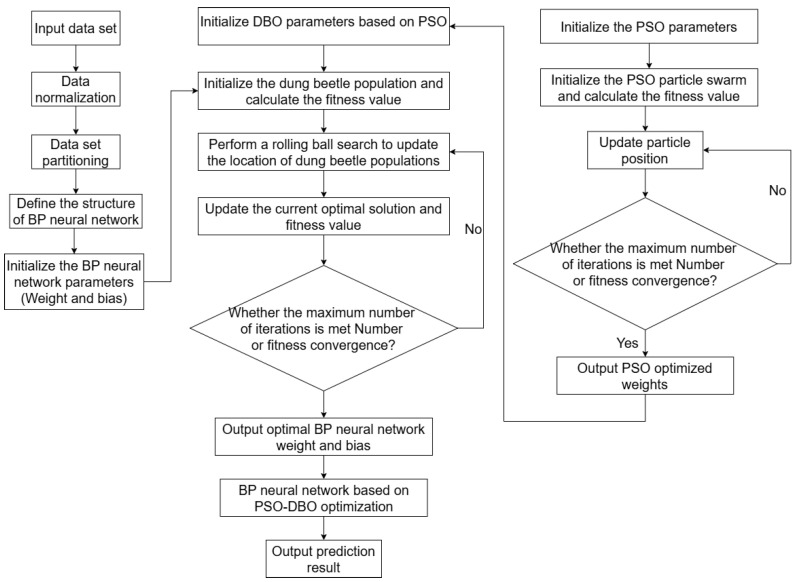
Overall flow chart of PSO-DBO-BP algorithm.

The BP neural network optimized by PSO and DBO can effectively predict the concentration of combustible gas and provide accurate gas detection results for practical applications. The overall flow of the PSO-DBO-BP algorithm is shown in Figure 3.

## 4. Experimental Tests

The neural network adopts a compact three-layer architecture, consisting of four input neurons corresponding to the gas sensor array, ten hidden neurons activated by the sigmoid function, and four output neurons with linear activation representing the concentrations of the four target gases. This configuration yields around 90 trainable parameters, including weights and biases.

The model was implemented using MATLAB 2021b. For a training dataset of 70 group samples over 100 epochs, the training process converged within approximately 19 s. The final trained model occupies approximately 0.8 KB in memory.

In terms of runtime performance, the average inference latency was measured to be less than 240 milliseconds per sample. These characteristics demonstrate the efficiency and deployability of the proposed system in wireless sensor networks and embedded environments.

**Figure 4:**
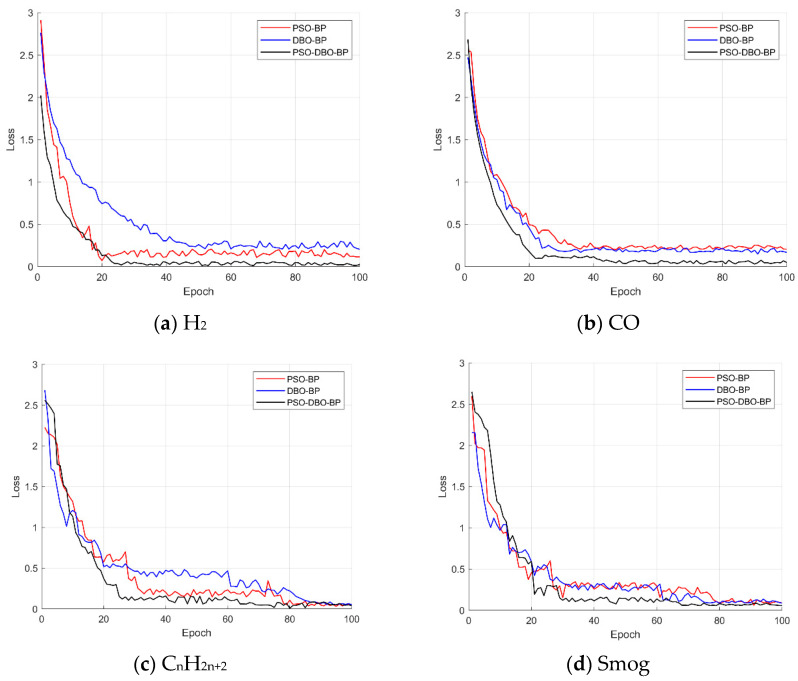
Variation trend of loss function of different optimization methods.

In order to evaluate the performance of different optimization methods in combustible gas detection tasks, three methods, PSO-BP, DBO-BP, and PSO-DBO-BP, were used in this study to train and test the data of four gas sensors. In this paper, the decreasing trend of the loss function MSE is used as the evaluation criterion to analyze the convergence speed and final accuracy of different methods. Figure 4 shows the variation trend of the loss function of the three optimization methods in the four gas sensor data regression tasks, where the abscissa represents the number of training rounds (Epoch), and the ordinate represents the loss value (Loss).

It should be noted that although 100 group data samples were collected in total, only 70 of them were used to train the models shown in Figure 4, while the remaining 30 were reserved for validation. The loss curves reflect the convergence behavior of each method over this 70-sample training set.

Figure 4 shows the variation trend of the loss function with the training rounds of three optimization algorithms (PSO-BP, DBO-BP, and PSO-DBO-BP) in four types of gas concentration detection tasks. On the whole, all the methods showed a faster convergence rate in the early stage, but the final performance on different datasets was different. In the detection tasks of carbon monoxide and smog gas, DBO-BP is much better than PSO-BP, indicating that DBO is more suitable for optimizing low-concentration gas data, which usually has weak features and high noise, and relies more on the local mining ability of the algorithm. However, in the detection of hydrogen and alkane gases, PSO-BP has a more prominent performance. These two types of data belong to high-concentration samples with clear feature distribution, and the global search mechanism of PSO is easier to obtain better solutions in such tasks.

The comprehensive comparison shows that PSO-DBO-BP finally achieves the optimal convergence performance in all tasks, taking into account the optimization requirements under different concentration characteristics. By integrating the global search capability of PSO and the local optimization characteristics of DBO, it can effectively overcome the limitations of a single optimization method, markedly improve the adaptability and robustness of the BP neural network in various gas detection scenarios, and improve the prediction accuracy of the neural network.

**Figure 5:**
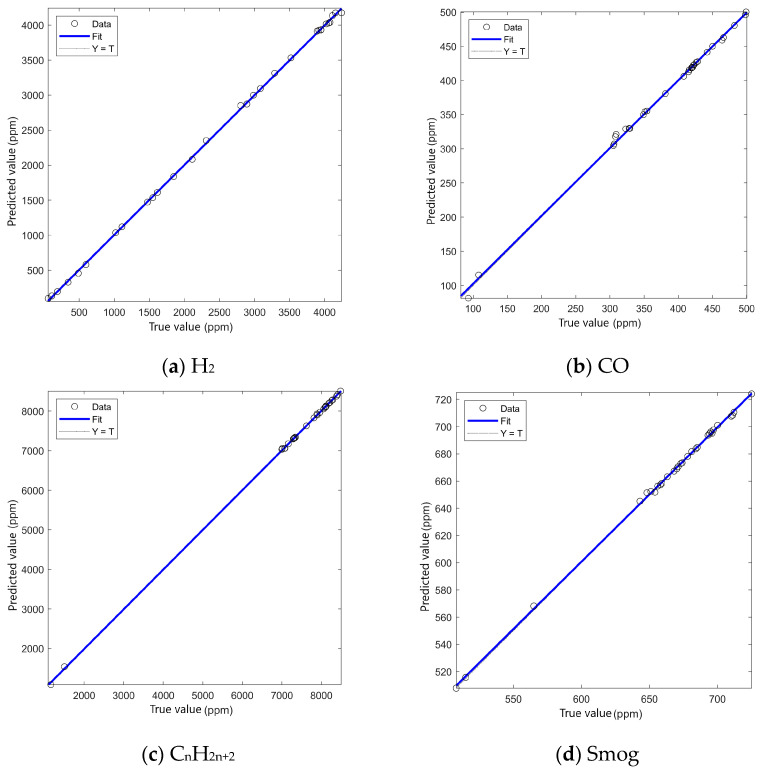
Regression analysis of four PSO-DBO-BP neural networks.

In order to further verify the fitting effect of this method in combustible gas detection tasks, the relationship between the predicted value and the true value is analyzed by regression. Figure 5 shows the fitting curve on the four sensors’ data of the neural network, where the horizontal coordinate is the true concentration (ppm) and the vertical coordinate is the concentration predicted by the neural network (ppm). In the figure, the blue line represents the ideal fitting curve (Y = T), the black circle represents the experimental data, and the blue broken line represents the fitting result of the neural network.

It should be noted that the CO gas concentrations used in the experiments range from 100–500 ppm, which are above the maximum safe exposure level for humans. These concentrations were selected to test the system’s robustness in detecting hazardous levels of CO in emergency scenarios, such as gas leakage or industrial emissions. The goal was to optimize the system’s performance under controlled but challenging conditions, where the system can detect CO leaks early before they reach dangerous concentrations.

Through the above analysis, it can be seen that the PSO-DBO-BP neural network shows a high degree of fit in all the regression tasks of sensor data, and the predicted value is highly consistent with the real value, indicating that the method can effectively learn the nonlinear relationship between gas concentration and sensor response. Compared with the single optimization method, this method further improves the global search ability of the model through the joint optimization mechanism of Particle Swarm Optimization and Dung Beetle Optimization, avoids the local optima problem, and enables the neural network to fit the data more accurately in the later training period. In summary, the regression effect of the PSO-DBO-BP neural network is much better than that of a single optimization method, and it can be applied to different types of combustible gas detection tasks.

In order to more intuitively compare the regression effect of the trained gas under different algorithms, the correlation coefficient R and the average relative error MRE after the training of DBO-BP are summarized in Table 2. The regression correlation coefficient R reflects the linear correlation between the predicted value and the true value, and the closer R is to 1, the better the regression effect is. The average relative error MRE represents the relative size of the prediction error, and the smaller the MRE, the higher the prediction accuracy.

By analyzing the data in the above table, it can be found that the data fusion effect of the PSO-DBO-BP neural network for multi-gas sensors is better than that of PSO-BP and DBO-BP, and its regression correlation coefficient (R) is much higher than that of PSO-BP and DBO-BP, and the average relative error (MRE) is lowest. The average relative error is further reduced and the correlation is improved, indicating that the prediction accuracy and stability of this method are better than that of a single optimization method in combustible gas detection tasks. Compared with PSO-BP, PSO-DBO-BP effectively overcomes the limitation of a single optimization method by combining the global search capability of PSO with the local optimization capability of DBO, so as to obtain the optimal solution in the detection task.

**Figure 6:**
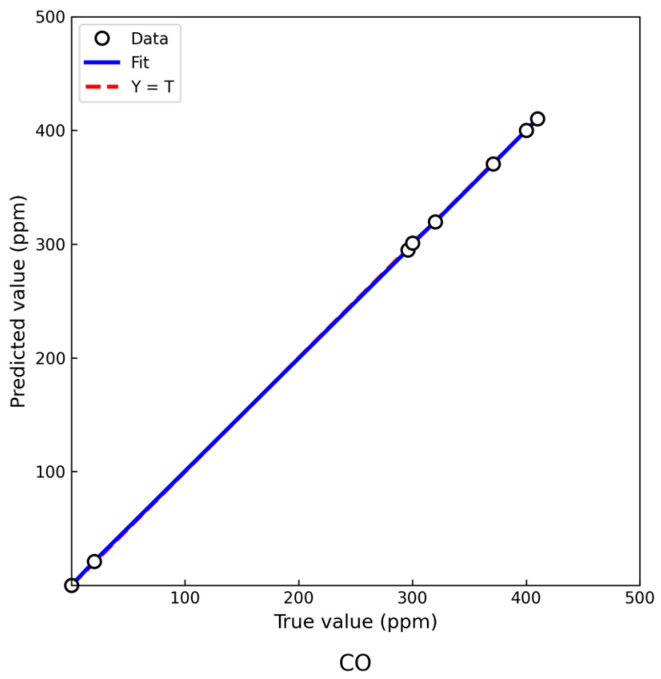
Regression fit between predicted and reference CO concentrations during smog exposure.

While Figure 5 illustrates the static regression performance across four gases using the proposed PSO-DBO-BP model, it is also essential to evaluate the model’s ability to track gas concentration changes over time. To this end, we conducted an additional smog exposure experiment and monitored the predicted and reference CO concentration values at several time points. Figure 6 presents this temporal comparison, using CO as a representative gas due to its relatively smooth variation and relevance in safety-critical applications.

The regression fitting results for CO gas concentration prediction are shown in Figure 6. Validation with actual sample data indicates a high degree of agreement between predicted and true concentrations, with most data points closely aligned with the ideal fitting line (Y = T). This suggests that the model exhibits strong linear fitting capability. Experimental results further demonstrate that the proposed neural network model can effectively capture the nonlinear relationship between CO concentration and sensor output, achieving high prediction accuracy and stability. These results confirm that the model meets the practical requirements of combustible gas detection tasks.

CO was selected as the representative gas because its concentration changed smoothly during the smog simulation, making it suitable for temporal evaluation. The predicted values consistently followed the trend of the reference values obtained from a calibrated detector. The absence of significant delays or deviations supports the model’s ability to track concentration dynamics, reinforcing its potential for time-sensitive environmental monitoring applications.

## 5. Discussion

### 5.1. Evaluation of the Proposed Hybrid Optimization Strategy

The focus of this study is to investigate the cooperative optimization potential of PSO and DBO when jointly applied to BP neural network training. Therefore, we limited our comparison scope to the two ablated variants, PSO-BP and DBO-BP, to clearly observe the performance gains introduced by the hybridization strategy.

PSO is widely recognized for its ability to rapidly explore the global search space and locate promising initial regions. However, it often suffers from premature convergence. DBO introduces adaptive multi-stage behavior that enhances local search accuracy and convergence stability. In the proposed model, PSO is used to provide high-quality initial parameters, and DBO performs targeted fine-tuning, effectively compensating for each other’s limitations.

Experimental results support this complementary mechanism: PSO-DBO-BP consistently achieves higher prediction accuracy, faster convergence, and more stable performance across different gas types. While this study did not include other optimization algorithms (such as GA, WOA, GWO, or DE) for comparison, our intent was to validate the benefits of this particular hybrid combination rather than to compare with a wide range of metaheuristic alternatives.

We acknowledge that broader comparative analysis would strengthen the generality of our findings and intend to incorporate it in future work. A more comprehensive set of experiments with GA-BP, WOA-BP, and other hybrid variants is already planned for subsequent studies.

### 5.2. Contribution of Sensor Design to Model Robustness

Another important aspect of the system’s effectiveness lies in how the multi-sensor array interacts with the model to support robust concentration prediction. Although each sensor in the array is primarily designed to detect a specific gas species, their responses often exhibit cross-sensitivity to other gases, an inherent characteristic of metal oxide semiconductor (MOX) sensors. This indirect signal carries latent information that can be learned by the neural network.

### 5.3. Modeling Performance Under Simulated Mixed-Gas Conditions

To better reflect real-world deployment scenarios, the experimental protocol was carefully designed to introduce multi-gas environments. The test chamber contained coexisting gases generated through combustion and leakage simulations, creating complex, cross-interfering atmospheres. These mixed-gas conditions induced nonlinear and overlapping sensor responses, which were used to train the PSO-DBO-BP model. The model successfully learned to infer true gas concentrations from fused sensor signals, as evidenced by the high regression correlations and low relative errors across all four gas types.

While these results are promising in controlled indoor settings, future research will further explore the model’s generalization to open-air, multi-source environments.

Through training on mixed-gas data, the PSO-DBO-BP model appears to extract complex nonlinear patterns from the combined sensor signals, allowing it to estimate concentrations even when a given sensor is not explicitly tuned to that gas. This capability is particularly advantageous in real-world environments where multiple gases co-exist.

### 5.4. Potential for Enhancing Model Interpretability via XAI

Building on this observation, it becomes important to understand how the model leverages such cross-sensitive features. While this hypothesis was not formally verified in this study, we acknowledge the importance of increasing the model’s transparency and interpretability. To address this, future work will incorporate explainable AI (XAI) techniques such as SHAP (SHapley Additive Explanations) and LIME (Local Interpretable Model-agnostic Explanations), which will help reveal feature contributions and interaction effects across sensors. This addition is expected to enhance the system’s interpretability and trustworthiness, particularly in safety-critical applications.

### 5.5. Identified Limitations and Future Research Opportunities

Despite promising experimental outcomes, several limitations of the proposed system should be carefully considered. While the proposed PSO-DBO-BP method demonstrates strong prediction performance in laboratory settings, several limitations of the system should be acknowledged. First, the model was trained and tested on data collected under controlled indoor conditions; its generalization to outdoor or industrial settings with varying humidity, temperature, or background interference has not yet been validated. Second, although the multi-sensor array can infer multiple gas concentrations through cross-sensitivity, this mechanism may become less reliable in more complex mixtures or environments with unknown interfering gases. Third, the current system does not yet incorporate real-time error detection or confidence estimation, which may be necessary for deployment in safety-critical applications. Lastly, energy efficiency, transmission latency, and long-term drift of the MOX sensors were not the focus of this study but will be addressed in future deployment-scale evaluations.

These limitations highlight key areas for future work and provide valuable context for interpreting our findings. Future work will also consider integrating additional error metrics such as Mean Squared Error (MSE) and Root Mean Squared Error (RMSE) to provide a more comprehensive quantitative evaluation of model performance.

### 5.6. Strategies to Prevent Overfitting with Limited Data

In addition to hardware and modeling concerns, attention was also paid to preventing overfitting during model training. Furthermore, the prevention of overfitting was also a key concern in model development, particularly given the limited size of the experimental dataset. Several techniques were adopted to mitigate overfitting risks:

(1) The dataset was partitioned into 70% training and 30% validation subsets, ensuring performance evaluation on unseen samples.
(2) The BP network architecture was deliberately kept shallow and compact to avoid unnecessary complexity.
(3) During training, validation loss was monitored to select the optimal stopping point and avoid over-training.
(4) The use of multi-dimensional inputs from cross-sensitive sensors implicitly introduced feature redundancy that may serve as a natural regularizer.

These measures helped maintain good generalization without compromising prediction accuracy. In future work, we aim to integrate more formal regularization techniques and cross-validation frameworks to further enhance the model’s robustness.

### 5.7. Comparative Performance with Related Studies

To further position our approach within the context of prior work, a comparative analysis with recent studies is provided. In addition to internal ablation comparisons, it is also important to situate the proposed method within the broader context of related studies. Several recent works have explored the application of neural networks and optimization algorithms in gas concentration estimation tasks. For instance, ref. [50] proposed a CNN-based method for mixed-gas detection on a small dataset, achieving efficient learning with limited training data while maintaining acceptable prediction accuracy. Ref. [51] combined PSO with CNN models using dynamically modulated temperature sensor data, resulting in improved concentration prediction and lower computational cost compared to traditional single-method approaches.

While deep models like CNNs and LSTMs are effective for tasks involving spatial or temporal feature extraction, they often require more training data and computational resources. In contrast, the proposed PSO-DBO-BP framework emphasizes lightweight regression-based inference suitable for deployment on microcontrollers and embedded platforms. Compared to existing approaches, our method achieved strong performance across four types of gases, with average mean relative errors below 0.25% and correlation coefficients exceeding 0.99, as reported in Table 2. The final model occupies only around 0.8 KB and provides predictions within 240 milliseconds per sample, validating its feasibility for edge implementation in wireless sensor networks.

Nonetheless, we acknowledge that a more comprehensive benchmarking particularly against other hybrid optimization algorithms such as GA-BP, WOA-BP, and GWO-BP, as well as LSTM and transformer based models, would provide additional insight into the generalizability and competitiveness of our method. Future work will include extended evaluations on larger public datasets and under varied environmental conditions to further support comparative analysis. These future efforts will be critical for validating the scalability and real-world applicability of the proposed system across varied deployment scenarios.

## 6. Conclusions

This study proposed a hybrid PSO-DBO-BP algorithm for improving the accuracy and stability of gas concentration prediction in wireless sensor systems. The method sequentially integrates Particle Swarm Optimization (PSO) for global parameter initialization and Dung Beetle Optimization (DBO) for adaptive local refinement, enhancing the training efficiency of the BP neural network. Experimental results under mixed-gas conditions demonstrate that the proposed model achieves high prediction accuracy, with average relative errors below 0.25% and correlation coefficients exceeding 0.99 across all tested gas types.

In addition to the algorithmic enhancement, the system integrates a low-power hardware platform based on Zigbee-enabled MOX sensor nodes, supporting real-time data transmission and distributed sensing. The model’s robustness in handling sensor cross-sensitivity and nonlinear gas interactions further contributes to its applicability in multi-gas environments.

Despite these promising results, the current study is limited to controlled laboratory settings. Generalization to outdoor or industrial scenarios, the integration of uncertainty estimation mechanisms, and benchmarking with other advanced optimization frameworks remain open areas for future exploration. The adoption of explainable AI tools such as SHAP and LIME is also planned to further improve model transparency and interpretability, especially in safety-critical applications.

Overall, the proposed PSO-DBO-BP approach provides a lightweight, adaptive, and hardware-compatible framework that can support accurate gas monitoring in embedded sensor networks. Future studies will focus on expanding its applicability, testing under real-world deployment conditions, and integrating more comprehensive comparative evaluations.

## Figures and Tables

**Table 1:** Sensor array gas concentration output data.

Number	H_2_ (ppm)	CO (ppm)	C_n_H_2n+2_ (ppm)	Smog (ppm)	Time
1	0	0	0	300	14 January 2025 20:43:07
2	12	48	62	386	14 January 2025 20:43:08
3	86	68	78	437	14 January 2025 20:43:08
4	110	96	1497	508	14 January 2025 20:43:09
…	…	…	…	…	…
50	2442	393	7736	620	14 January 2025 21:30:00
51	2531	396	7756	623	14 January 2025 21:30:01
52	2617	401	7762	626	14 January 2025 21:30:01
…	…	…	…	…	…
99	4166	494	8402	720	14 January 2025 23:00:00
100	4215	500	8484	725	14 January 2025 23:00:01

**Table 2:** Regression correlation and error analysis.

Gas	PSO-BP		DBO-BP		PSO-DBO-BP	
	R	MRE	R	MRE	R	MRE
H_2_	0.9912	0.88%	0.9821	1.79%	0.9984	0.16%
CO	0.9850	1.50%	0.9902	0.98%	0.9978	0.22%
C_n_H_2n+2_	0.9863	1.36%	0.9881	1.19%	0.9986	0.14%
Smog	0.9907	0.93%	0.9819	1.81%	0.9992	0.08%

## Data Availability

The data used to support the findings of this study are available from the corresponding author upon request.
